# Gender Differences in Material, Psychological, and Social Domains of the Income Gradient in Mortality: Implications for Policy

**DOI:** 10.1371/journal.pone.0059191

**Published:** 2013-03-20

**Authors:** Peter Muennig, Meghan Kuebler, Jaeseung Kim, Dusan Todorovic, Zohn Rosen

**Affiliations:** 1 Department of Health Policy and Management, Mailman School of Public Health, Columbia University, New York, New York, United States of America; 2 Department of Health Policy and Management, Mailman School of Public Health, Columbia University, New York, New York, United States of America; 3 Mailman School of Public Health, Columbia University, New York, New York, United States of America; Old Dominion University, United States of America

## Abstract

We set out to examine the material, psychological, and sociological pathways mediating the income gradient in health and mortality. We used the 2008 General Social Survey-National Death Index dataset (N = 26,870), which contains three decades of social survey data in the US linked to thirty years of mortality follow-up. We grouped a large number of variables into 3 domains: material, psychological, and sociological using factor analysis. We then employed discrete-time hazard models to examine the extent to which these three domains mediated the income-mortality association among men and women. Overall, the gradient was weaker for females than for males. While psychological and material factors explained mortality hazards among females, hazards among males were explained only by social capital. Poor health significantly predicted both income and mortality, particularly among females, suggesting a strong role for reverse causation. We also find that many traditional associations between income and mortality are absent in this dataset, such as perceived social status.

## Introduction

In the US, those in the lower 80% income bracket lose 17.4 million years of perfect health per year relative to those in the highest 20% of income earners. [1] This amounts to approximately 361,000 (15%) of all deaths in the US, [1] and is associated with more than twice the number of quality-adjusted life years lost to obesity. [2] This association between wealth and health weakens as incomes rise, but some association remains even when comparing relatively wealthy populations to slightly less wealthy ones. Because this association is graded, it is often referred to as the health-wealth “gradient” [3], [4].

It is called a gradient because the higher a given group’s income, the lower the chances that the group will be exposed to a wide array of risk factors for poor health outcomes. These risk factors for premature mortality can be roughly grouped into 3 larger categories: material, psychological, and social. [3], [5]–[9] Within each of these categories, low income groups might be exposed to a number of health threats, some small and some large, that cumulatively add up to a much higher risk of death overall.

For instance, material pathways to premature mortality might include the inability to purchase high quality housing, afford to live in a low-crime neighborhood, or afford healthy foods. [10], [11] Social pathways to premature mortality are often broadly grouped into a concept called “social capital.” Social capital takes many forms, but very generally refers to those features of social relationships such as interpersonal trust, norms of reciprocity, and mutual aid. [12], [13] Social capital appears to increase with income possibly in part because intact families tend to have higher earnings and partly because income is associated with the financial resources needed to socialize with others who are well connected (e.g., to good jobs or skilled doctors). [14] Psychological pathways linking a higher income to a longer life include: psychological stress, positive emotional states (such as happiness), or differences in perceived social standing.

Because psychological pathways are not intuitively linked to income or to health for most readers, they require some elaboration. First, despite perceptions that high-income populations experience significant psychological stress in the workplace, surveys show that lower income populations report higher levels of psychological stress both at home and at work. [15] Psychological stress is hypothesized to increase one’s risk of premature mortality by producing disruptions in neuroendocrine systems in the body, leading to oxidative damage that causes premature aging. [7], [16]–[18] Some research suggests that symbolic resources, such as control, prestige, and social status, can also increase one’s risk for poor health outcomes. [13], [19] That is, being lower on the social totem pole can produce psychologically stressful status anxiety that, in turn, leads to higher mortality. Positive psychological states, such as happiness, a happy marriage, and satisfaction with one’s family and leisure time are believed to increase longevity, possibly by reducing psychological stress [7], [16], [19]–[21].

However few such factors have been proven to be causal, in part because there are relatively few randomized experimental studies on humans to test the role of specific material, psychological, and sociological factors as mediators in the income gradient. [22]–[24] In the absence of experimental studies, it is important to examine putative mechanisms underlying the income gradient using a single dataset. [10], [25]–[29] This way, correlational research can guide policy experiments that focus on specific mechanisms. This is a non-trivial point, as these experiments cost many millions of dollars to conduct and the policies that they support tend to have costs in the tens of billions of dollars in large industrialized nations.

When individual mechanisms are explored via many different sources of data, publication bias may elucidate pathways that are, in fact, attributable to random observations. [30], [31] That one’s thoughts or perceptions play a role in explaining the income-mortality gradient is of great interest to many researchers. [32]–[37] However, it is not surprising (and thus not very interesting) if our thoughts are not in fact making us more sick. Thus, a positive finding is highly likely to be published in the scientific literature, and a negative finding is highly unlikely to be published, leaving only positive findings in the literature.

Finally, it is important to have access to the variables needed to explore the influence of reverse causality and confounding in the gradient. Those who become sick are also likely to lose their job and incur medical costs, thus becoming poorer. [3], [38] Moreover, reverse causality can also apply to mediators; while income might make people happy, happy people may also be better at studying for college, securing a job, and being promoted at work, as compared to sad people. [39] Healthy people might be both happier and wealthier as a result of their health. Intergenerational transfers of health and wealth could also play a large role. [40] That is healthy parents tend to be wealthier as a result of their health and healthier parents may be more likely to have healthy children. These factors might all be lumped under the category of measurement error. That is, if our objective is to measure forward causality to inform redistributive policies, then reverse causality and confounding produce systematic error that biases the estimates of any benefits that might be realized by such policies.

Reverse causality can be addressed by including only participants who report that they are happy or in good health at the time of the interview, or by controlling for these factors. [41] Likewise, parental characteristics can be held constant. If parental characteristics play a large role, then policies targeting adult income redistribution are unlikely to improve health, but effective school reform might.

In this paper we attempt to dissect the income gradient using the wide range of variables available in a single dataset that contains material, psychological, and social measures as well as methods for ascertaining the influence of reverse causality or intergenerational transfers of health and wealth. This paper contributes to the literature by: 1) exploiting a single dataset, thus allowing for examination of multiple pathways at once, 2) by exploring the relative contribution of each group of factors separately by gender, 3) by addressing major sources of confounding that are not normally available, 4) by using a dataset with long-term mortality follow up (a potential source of bias in most datasets). We do this using the General Social Survey-National Death Index, which allows for estimation of the relationship between income and mortality rates in a representative sample of the civilian (non-institutionalized) US population that contains 30 years of mortality follow-up data.

## Materials and Methods

### Data

Our analysis was performed using the 2008 General Social Survey-National Death Index (2008 GSS-NDI) dataset, which links the 1978–2002 waves of the GSS to NDI data through 2008. [42] The 2008 GSS-NDI provides three decades of data that can be weighted to be representative of the US (non-institutionalized) civilian population. It includes a total of 32,830 participants, of which 9,271 were deceased as of 2008.

After removing those who were foreign-born, and those with missing data on income, age, gender, race, and/or geographic region, 26,870 participants remained. Foreign-born subjects were dropped from the sample because selection appears to confound the income-mortality relationship in this group. In analyzing the influence of material, psychological and social domains on the income-mortality rate gradient, the sample sizes change slightly because some variables were not obtained in particular waves of the GSS. However, because each sample is nationally-representative, this should not affect the overall representativeness of our sample.

### Data Availability

We previously published a manuscript describing the GSS-NDI data and how it can be downloaded. [42] The data can be directly accessed at http://www3.norc.org/GSSWebsite/Download/.

### Measures

Our principal outcome of interest was mortality hazards. Our primary independent variable was inflation-adjusted family income standardized to year 2000 dollars (this is a variable available in the GSS-NDI based on adjustments made using the Consumer Price Index). [42], [43] This measure includes all income received annually by family members, including wages, capital income and taxes. From this standardized measure of income we created income quintiles to account for the non-linear association between income and mortality hazards. We used quintiles because they provided the smallest intervals for which we still had adequate power to detect differences in mortality at p<0.05 and power of 0.8. We explore mechanisms within the first quintile (to examine the effect of material deprivations) as well as quintiles 2–4, to examine why the middle-quintile income earners experienced higher mortality hazards than the highest quintile. The income quintiles were grouped as follows: Quintile 1: $480 to $15,700, Quintile 2: $15,701 to $28,400, Quintile 3: $28,401 to $44,000, Quintile 4: $44,001 to $67,400 Quintile 5: $67,401 and above.

We defined age, race, gender, educational attainment and survey year of the interview as baseline control variables in the income-mortality rate association and included them in all models. We controlled for age, race, and gender to capture fixed socio-demographic characteristics. The educational attainment of the adult participants was included as a confounder rather than a mediator because: 1) education is known to be independently predictive of adult health and higher income [44], [45], 2) while a child’s parental income is highly correlated with the child’s subsequent educational attainment, [46] our models account for parental educational attainment (see below), and 3) it is unlikely that a large number of adults with higher earnings used their higher adult earnings to purchase additional income. Survey year was included to capture period effects.

We next defined other characteristics as potential explanatory variables, and examined their influence on the gradient independently. Specifically, we explored; 1) material factors (owning a house vs. renting house), 2) psychological factors (overall happiness, marital happiness, subjective perception of socio-economic status, and satisfaction with friends, job, family, and hobby), and 3) social characteristics as follows: a) social capital (trust in others, feeling that people look out for themselves), b) social support (spending time with friends, relatives, and family), and c) religious activity (frequency of attending religious services, frequency of prayer, and strength of religious affiliation). The measures were selected to capture dimensions (e.g., social capital) within each domain (e.g., social), with the recognition that many of these characteristics potentially overlap. Then, explanatory factor analysis was performed to reduce the dimensionality of each domain. Varimax rotation was applied to supply the data structure, and the scree test was used to retain factors. [47] The analysis identified housing tenure as the lone measure of material circumstances; subjective perception of socio-economic status (satisfaction with financial situation and subjective assessment of financial situation relative to average, factor loadings ranging from 0.53 to 0.61); existential satisfaction (overall happiness, happiness with marriage, and satisfaction with job, factor loading ranging from 0.33 to 0.58); satisfaction with leisure time (friends and hobbies, factor loadings ranging from 0.40 to 0.41); bridging social capital (trust in others, feeling that people look out for themselves, factor loadings ranging from 0.48 to 0.49); frequency of contact with friends (factor loadings ranging from 0.41 to 0.43); frequency of contact with family (factor loadings ranging from 0.63 to 0.68); and frequency of involvement in religious activities (factor loadings ranging from 0.61 to 0.73).

### Statistical Analyses

As our data measured time discretely, we used discrete time hazard models to predict hazard ratios among income intervals. Discrete time hazard models estimate the proportion of the sample who experience the event (in this case, death) during a specific time period (1979–2008). [48] For our analysis, individual cases were expanded into annual records over the 30-year duration of follow up. Our dependent variable is the vital status of the individual within a given year, which is dichotomous in nature.

We evaluated linear, quadratic and higher order polynomial specifications of time to determine the most parsimonious functional form, and chose the quadratic form as it provided the best model fit. We calculated hazard rates using the complementary log-log link because the Cox Proportionate Hazards models failed proportionality assumptions. This technique has the advantage of being comparable to the Cox proportional hazard in continuous time, since the exponentiated coefficients from a discrete-time hazard model with the cloglog link are able to be interpreted as a hazard ratio. [49] The cloglog discrete-time hazard rate *h* for individual *i* is:
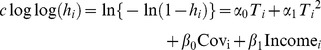
where: T_i_ indicates the time since survey and T^2^_i_ is a quadratic time term that captures a non-linear trend of time; Cov represents a vector of covariates at the survey year and *Income* is quintile income at the survey year. To test the proportional hazard assumption we first examined the interaction of duration of survival and quintile income, which was significant as expected. Then, we plotted the log-log survival curves for each level of income. The vertical differences between curves were approximately equal throughout the follow-up period of 30 years, indicating that there was evidence of proportionality in hazard ratios of our model.

The explanatory variables of interest are plausibly linked to higher income. To test the influence of our constructed domains on the income-mortality hazards relationship, we employed the traditional mediation approach of Baron and Kenny using combined item responses (treated as a single continuous variable). [50], [51] While the Baron and Kenny approach is not technically ideal for a survival model, it does provide the reader with a sense of the overall impact of each mediator domain on the income gradient in mortality and serves as a very conservative estimator of mediation. First, we examined the relationship between income and mortality hazards for each quintile stratified by gender as a baseline model. We then tested the relationship between income quintiles and each explanatory mediating variable under study. We next tested the relationship between the explanatory mediating variables and mortality hazards by adding mediators in the baseline model. This model is:
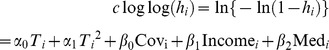
where Med_i_ indicates each mediating variable for individual *i*.

Finally, we examined whether adding explanatory mediating variables reduce the total effect of income on mortality by measuring changes in the hazard ratio (ΔHR). Potential psychosocial and material explanatory variables were added one at a time to the baseline model, and were tested in separate models. We repeated this process testing each potential explanatory variable as a mediator between the income and mortality.

### Sensitivity Analyses

To explore the effect of reverse causality in the gradient, we included self-rated health and self-rated happiness as control variables. We also explored whether self-rated health and happiness played meditational roles (e.g., higher income leads to higher happiness which in turn leads to better health). We also limited the analyses to participants in good or excellent self-rated health and participants who reported being pretty happy or very happy, but we did not have sufficient sample sizes to detect effect sizes smaller than a hazard ratio of 1.8 (at a power of 0.8 with p<0.05), and therefore did not include these analyses.

The father’s educational credentials serve as a predictor of the participant’s future education and income well in advance of any potential for sickness to influence health. We therefore next ran the analyses three ways to tease out the effect of using the father’s highest educational degree. (We chose father’s highest educational degree, as the GSS-NDI data go back to 1978 when fewer women were in the workforce.) First, we ensured that father’s highest degree was predictive of mortality. The next model included both income and the father’s highest degree. This helps account for the intergenerational transfer of health as described in the introduction. The third model controlled for income and both the father’s highest degree and the participant’s highest degree. This helps control for the intergenerational transfer of education credentials.

The GSS-NDI was approved by the Columbia University Institutional Review Board.

## Results

**Figure 1:**
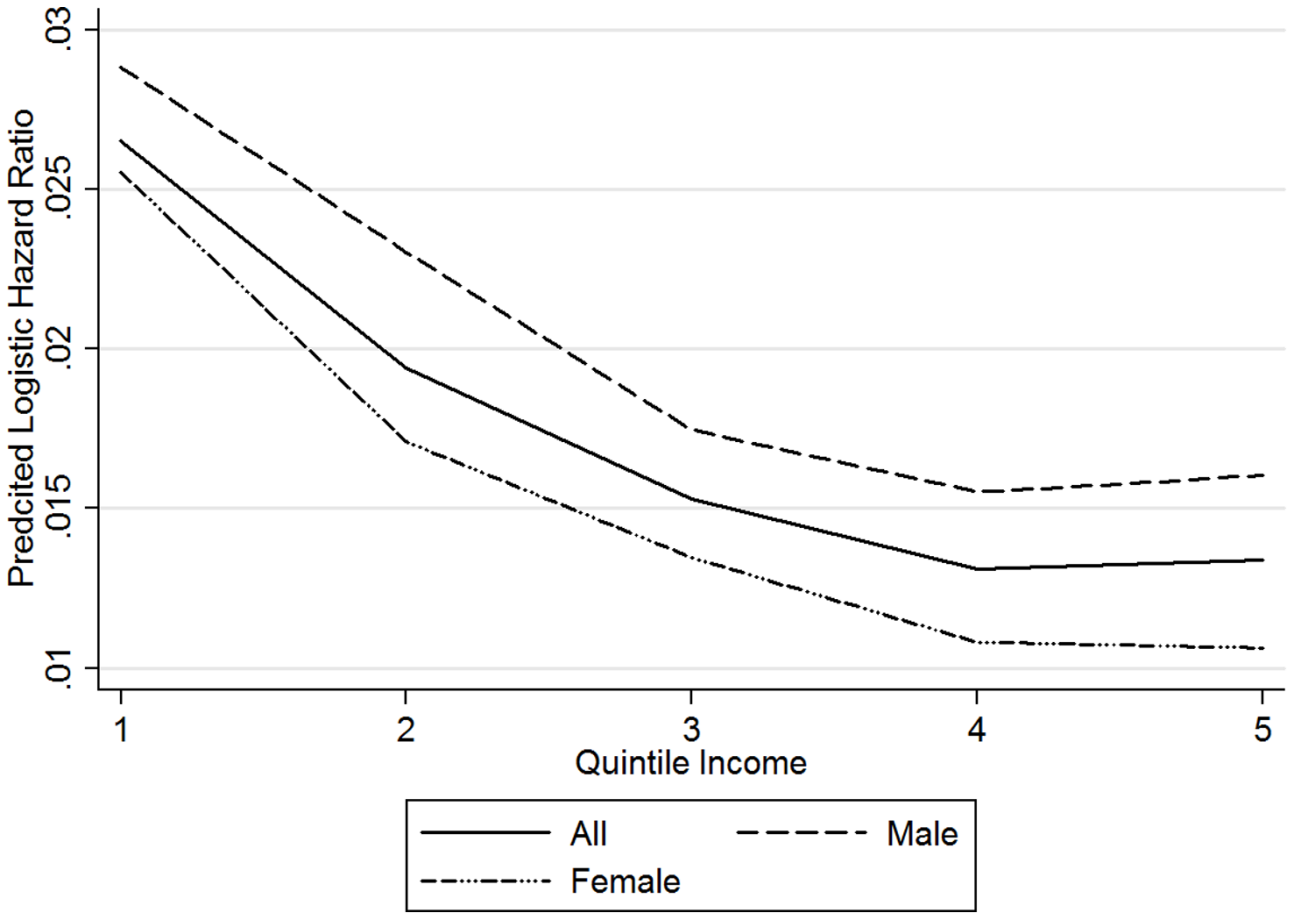
Hazard Ratio by Quintile Income for Total Population. 1978–2002 General Social Survey linked to 2008 mortality data via the National Death Index.

We observed a curvilinear relationship between mortality risk and the bottom 80% of income earners (**Figure 1**). However, the relationship was weaker for females than for males. **Table 1** shows the demographic characteristics of our overall sample by gender.

**Table 1:** Demographic characteristics of the analytic sample.

	Male	Female
N	11,866	15,004
Age (%)		
Under 25	12.9	12.4
25–34	23.9	24.2
35–44	22.0	20.2
45–59	22.0	20.6
60–69	10.9	11.3
70 and over	8.3	11.3
Race (%)		
White	87.4	83.7
Black	10.1	13.9
Other	2.4	2.4
Education (%)		
Less than high school	21.3	22.0
High school graduates	29.9	33.9
Some college	24.3	24.5
More than college	24.5	19.6
Income (constant Yr. 2000 $)Mean (SD)	48,468 (35,092)	40,232 (33,169)

1978–2002 General Social Survey linked to 2008 mortality data via the National Death Index.

**Table 2** shows the main effects of income on mortality hazards stratified by gender. The fifth quintile (highest earners) is held as the reference group. For men, the income gradient extended to the 60^th^ percentile of income earners. This was true even when controlling for health at the time of interview. For women, the income gradient was significant only among the two bottom quintiles, and even then only when baseline health is not considered.

**Table 2:** Adjusted hazard ratio associated with each income quintile by gender (standard error).

Income		All Subjects	Males		Females	
			Baseline	Baseline+Health	Baseline	Baseline+Health
Quintile 1	1.336***		1.367***	1.215*	1.248***	1.127
	(0.057)		(0.084)	(0.093)	(0.076)	(0.086)
Quintile 2	1.259***		1.336***	1.247**	1.172**	1.163*
	(0.051)		(0.076)	(0.088)	(0.071)	(0.087)
Quintile 3	1.126**		1.207***	1.158*	1.027	0.988
	(0.047)		(0.067)	(0.079)	(0.065)	(0.077)
Quintile 4	1.043		1.047	1.043	1.029	0.960
	(0.045)		(0.059)	(0.073)	(0.068)	(0.078)
Quintile 5	1		1	1	1	1
					
N	26,870		11,886	8,408	15,004	10,536

1978–2002 General Social Survey linked to 2008 mortality data via the National Death Index.

*** p<0.001,

** p<0.01,

* p<0.05.

Note: All models control for age, gender, race, survey year and educational attainment.

Income Quintile 5 is the reference group.

**Table 3** describes the influence of the variables under study in the relationship between income and mortality hazards for males and females. The first column describes the mediator domain. The remaining columns present the baseline hazard ratio (HR) for income on mortality among the sample of participants for whom the putative mediator was asked. The third column presents the HR for the baseline model plus the mediator domain listed in column 1. The next column presents the hazard ratio for the mediator domain itself. The final column presents the change in the HR when the mediator is added. While this approach is underpowered to detect mediation, it does provide the reader with a sense of the clinical significance of the variables as mediators. Self-rated health played a large role in explaining the income gradient (1.8% Δ in the HR for males and a 2.7% Δ in the HR for females; p<0.001 for both genders). Psychological factors and home ownership played a clear meditational role for females (1% Δ in the hazards ratio [HR]; p<0.001) and (2.6% Δ in the HR; p<0.001). Males with higher social capital also saw a reduction in hazards (0.4% Δ HR; p<0.05).

**Table 3:** Percentage change in hazard ratios associated with various material and psychosocial characteristics by gender (standard error).

Domain	N	Hazard Ratio forIncome on Mortality	Hazard Ratio for Income + Mediator	Hazard Ratio for Mediator	% Change in Hazard Ratio
**Males**					
Self-Rated Health	8,408	0.927***(0.016)	0.943***(0.016)	1.194***(0.030)	1.8
Material Wealth^a^	6,104	0.880***(0.018)	0.892***(0.019)	1.123(0.069)	1.2
Subjective Social Standing^b^	11,860	0.916***(0.013)	0.924***(0.014)	0.953(0.036)	0.8
Existential Satisfaction^c^	11,405	0.915***(0.012)	0.919***(0.013)	1.049(0.033)	0.4
Satisfaction with Leisure Time^d^	5,599	0.931***(0.016)	0.935***(0.016)	1.089(0.049)	0.4
Social Ties^e^	8,011	0.913***(0.015)	0.917***(0.015)	1.132*(0.058)	0.4
Structural Social Capital^f^	7,351	0.895***(0.016)	0.896***(0.015)	0.996(0.029)	0.1
Family Ties^g^	7,349	0.896***(0.016)	0.896***(0.016)	1.000(0.027)	0
Religious Community^h^	11,844	0.916***(0.013)	0.916***(0.013)	0.969(0.025)	0
**Females**					
Self-Rated Health	10,536	0.932***(0.016)	0.957*(0.017)	1.231***(0.030)	2.7
Material Wealth^a^	7,772	0.943**(0.020)	0.968(0.019)	1.236***(0.067)	2.6
Subjective Social Standing^b^	14,992	0.941***(0.013)	0.932***(0.014)	1.058(0.037)	−0.9
Existential Satisfaction^c^	14,381	0.940***(0.013)	0.950***(0.014)	1.117***(0.040)	1.0
Satisfaction with Leisure Time^d^	7,219	0.941***(0.016)	0.944***(0.017)	1.064(0.047)	0.3
Social Ties^e^	10,105	0.944***(0.016)	0.945***(0.015)	1.021(0.052)	0.1
Structural Social Capital^f^	9,477	0.946***(0.016)	0.946***(0.016)	1.059(0.034)	0
Family Ties^g^	9,473	0.917***(0.010)	0.917***(0.011)	0.994(0.02)	0
Religious Community^h^	14,971	0.942***(0.016)	0.942***(0.016)	0.976(0.025)	0

1978–2002 General Social Survey linked to 2008 mortality data via the National Death Index.

*** p<0.001,

** p<0.01,

* p<0.05.

All models adjust for Age, Gender, Race, Survey Year And Educational Attainment. The first formula (represented in column 3) controls only for these variables. The second formula controls for these variables plus the mediator and presents the coefficient for income when the mediator is added (column 4) and for the mediator (column 5).

a Rent or Own Dwelling.

b Subjective Assessment of Income Relative to Average; Satisfaction with Financial Situation.

c Happiness, Happiness with marriage, Satisfaction with Job.

d Satisfaction with Friends and Hobby.

e People Try to be Helpful; People Can be Trusted.

f Frequency of Time Spent with Friends.

g Frequency of Time Spent with Family.

h Frequency of Attending Religious Services or Practicing Prayer; Strength of Religious Affiliation.

In addition to including self-rated health as a covariate in the model (**Table 2**), we also restricted the sample to only those in good or excellent health (results not shown). This rendered all explanatory variables non-significant, but it also limited the power to detect effect sizes that were smaller than those observed in **Table 3**.

The participant’s father’s highest degree also showed a similarly strong gradient in mortality hazards. When parental education was included as a covariate in the model, income continued to predict mortality (HR = 0.95; 95% CI = 0.92–0.88). However, this gradient disappeared entirely when the participant’s own highest educational degree was also added to the model.

## Discussion

After the discovery of the income gradient in mortality rates, researchers sought to understand why it is that those in the mid-range of incomes–people who were not materially deprived–might be at higher risk of mortality than those who were wealthier still. [10] The leading hypotheses surrounded material, psychological and social factors in health. [3], [10] However, until the advent of the GSS-NDI, there was no good way of exploring the relative contributions of many of these domains within the gradient at the same time and few datasets were robust enough to control for most potential confounding variables or for reverse causality.

We find income is significantly associated with mortality hazards up through the bottom 60% of all households in the US. This association was much weaker for females than for males. Among females, being healthy, owning a home, and being existentially fulfilled (happy, happy with one’s marriage, and satisfied with one’s life, marriage, and job) proved to be the most important variables with respect to mediating premature mortality within the income gradient. For males, health and bridging social capital (trust in others and a belief that people try to be helpful) proved to be the only important mediator in reducing premature mortality within the income gradient.

When we re-ran the analysis stratifying by father’s highest degree rather than income quintile we found a similarly strong gradient in mortality rates as well. Adding the father’s highest degree to the income-mortality association only tempered the association. However, this gradient in the father’s educational credential and the association between income and mortality both disappeared when the participant’s own highest degree was added to the models. Taken together, these findings suggest that intergenerational transfer of education (but not necessarily wealth or health) is a powerful explanatory variable in the gradient. Early education policies may therefore be called for as public health measures intended to reduce the gradient (and thus health disparities by income).

Using factor analysis, happiness and life satisfaction variables converged into one domain in our dataset. Happiness is fairly well established in the literature as a correlate of mortality. [20] Previous studies on broader measures of well-being, such as happiness with marriage or life satisfaction, have shown mixed, but generally positive results as correlates of income and/or mortality. [52]–[54] One previous study found an association between general life satisfaction and mortality for men but not women–the opposite of the findings observed in our study. [52] However, many of these earlier studies controlled for covariates that are plausibly in the pathways through which existential angst may exert its influence on mortality: behavioral risk factors (e.g., dissatisfied people may be more likely to smoke than content people) and biological measures, such as cholesterol and blood pressure (e.g., anxious people may be more adrenergic and therefore have a higher blood pressure). [18] Rather than controlling for these risk factors, we hold health constant. This allows us to observe what happens to those who report being in good health at the time of the interview over many decades of follow up. While this approach is also conservative and eliminates some individuals, it still allows us to capture some of the influence of behavioral risk factors on later mortality. Social capital is generally thought to be beneficial for both genders [14].

Home ownership explained a relatively larger amount of variation in the hazards ratio as opposed to psychological or social factors, but this variation was only significant for females. Home ownership can be a measure of neighborhood qualities, total family wealth, or the forward-looking nature of the respondent. [5], [55] (That is, those who buy homes may be more future oriented than those who do not, and therefore less likely to engage in risky behaviors that could shorten one’s life).

Some of the findings were surprising. For instance, our composite measure of perceived social status was not significantly correlated with mortality hazards, and played only a small role in mediating the gradient. In fact, for women, one’s perception of her social status (as measured by her assessment of her income relative to ‘the average’ and her satisfaction with her financial situation) produced the opposite of the expected effect–actually widening the hazards of mortality in the gradient. Relative social status is believed by some to be a major explanatory variable in the income gradient, particularly among those with enough income to access all the material comforts that modern industrialized nations afford. [56], [57] However, it should be noted that our factor analysis of three variables is not an established measure of relative social status.

As might be expected, self-rated health played a role as a confounder (Table 2) and a mediator (Table 3). In fact, self-rated health explained all of the association between income and mortality hazards for females for the top three quintiles, and left only marginally significant effects for the bottom two quintiles (Table 2). In contrast to our study, the Alameda County study showed strong effects of income on health when selecting only healthy subjects for follow-up. [41] Moreover, the general patterns of mortality by age suggest that reverse causality plays less of a role in the gradient than we observe in the present study [58].

Our study has a number of important limitations. Foremost, we did not have the statistical power to stratify the analysis by self-rated health. Second, we do not have the statistical power to explore the relative contribution of risk factor categories over time. Third, we use a prospective cohort study to examine the associations rather than an experimental design. Therefore, the directionality of the effect of the putative explanatory variables under study is not testable and unobserved confounders could play a role in the meditational effects we observe [14], [22], [59].

Our study tests a large set of material, psychological, and sociological explanatory variables in the gradient in income and mortality rates using a single dataset. While most of effects of known material, psychological, and sociological variables were both positively correlated with income and inversely correlated with mortality as expected, remarkably few were statistically significant. This was true despite the fact that we had sufficient power to detect less than a one percent change in hazards of the overall association for most variables. Moreover, we find that women who have more money do tend to see their financial situation as better than others, but that this perception plays no role in the gradient. We conclude that, while the hypothesized material, psychological, and social factors are important explanatory variables, not all are important, and certainly not all are important equally for both genders.
